# Continuous Hand-Arm Vibrations Do Not Interfere with Cognitive Processing

**DOI:** 10.5334/joc.490

**Published:** 2026-02-17

**Authors:** Anne Voormann, Andreas Lindenmann, Jan Heinrich Robens, Sven Matthiesen, Andrea Kiesel

**Affiliations:** 1 Department of Psychology, University of Freiburg, Germany; 2 Institute of Product Engineering, Karlsruhe Institute of Technology, Germany

**Keywords:** hand-arm vibrations, cognitive processes, selective attention, (temporal) Flanker task

## Abstract

When humans engage in closely coupled human-machine interactions, they often experience hand-arm vibrations, which are a byproduct of the running machine. Yet, in closely coupled human-machine interactions, it is important to ensure that human attention and cognition remains sufficiently high to avoid accidents and to achieve a good performance. The aim of the present study was to examine whether hand-arm vibrations impact on cognitive processing. In two studies, we investigated the impact of constant or random vibration compared to a baseline condition without vibration on selective attention. In detail, we assessed overall performance (RT and error rates) and the congruency effect in a flanker task (Experiment 1) and a temporal flanker task (Experiment 2). In Experiment 2, we additionally explored experienced vibration comfort and discomfort, two constructs often considered in ergonomics. In both experiments hand-arm vibrations neither affected mean response times nor proportion of correct responses. Additionally, hand-arm vibrations did not modulate the congruency effect. Experiment 2 revealed that vibration comfort and discomfort seem to correlate with task-performance. We conclude that hand-arm vibrations in general do not impact on cognitive processing, but it seems important to consider which vibration is selected to achieve optimal performance depending on user experience.

Hand-arm vibrations often arise in closely coupled human-machine interactions, e.g., when using a power tool like a grinding or drilling machine or when holding a workpiece that is currently under workmanship. In these situations, it is important for craftsmen to be attentive while experiencing hand-arm vibrations to achieve a good working result and to avoid accidents or damages. These activities have in common that hand-arm vibrations cannot be avoided nor can these activities be replaced by automation. Although vibration-isolating handles can reduce the transmitted vibrations, it must be taken into account that vibrations can also serve as feedback for the user. In future, the machines or the vibration-isolating handles could be designed in such a way that the transmitted hand-arm vibration supports task performance and minimizes risks of accidents due to human failures. To this end, it is necessary to investigate which type of hand-arm vibrations possibly facilitates or interferes with cognitive performance, which is the aim of the present study.

So far, only few studies investigated the impact of vibration on cognitive performance. Most of these studies investigated whole-body vibration, which is used as a training method that stimulates the human neuromuscular system (Wen et al., 2023). However, evidence is mixed regarding its impact on cognitive task performance. While Regterschot et al. (2014) observed an improvement in performance in a Stroop task (Stroop, 1935), a color word interference task, after an exposure to whole body vibration of 2 min, they did not observe any impact in a working memory task. In contrast, Gritschmeier (2021) did not find a positive effect of a 4 min exposure to whole-body vibration on attention and concentration in complex tasks, on recall, or on inhibitory control. However, they observed that vibration frequency moderated the time of a mental rotation test with medium intensities leading to faster responses. While the previous studies have investigated cognitive processing after the exposure to whole-body vibration, Ljungberg et al. (2004) assessed the impact of whole-body vibration in combination with and without noise on short-term memory performance while being exposed to vibration and noise. Objectively measured short-term memory performance was not affected by vibration and noise. However, subjective task difficulty was increased in the condition combining vibration and noise compared to a condition without vibration and noise, a condition with only vibration, and a condition with only noise.

A further study investigated the impact of hand-arm vibrations experienced by bus drivers throughout a workday on Stroop task performance. It was observed that higher acceleration of hand-arm vibrations led to increased interference times (Rahmani et al., 2021). However, in this study cognitive task performance was only assessed after the exposure to hand-arm vibrations. In contrast, in many work contexts, such as those described in the introduction, a high level of attention and attentional focus is required *while* experiencing hand-arm vibrations. To address this research gap, we aim to compare cognitive processing, more precisely attentional focus, in conditions with and without hand-arm vibrations.

A common task to examine the attentional focus is the flanker task (B. A. Eriksen & Eriksen, 1974). In a flanker task, participants have to respond to the identity of a central stimulus (e.g., S), which is flanked either by distractors having the same identity (e.g., SSSSS – congruent trials) or by distractors having a different identity (e.g., KKSKK – incongruent trials). We selected the flanker task, as the size of the flanker congruency effect (response time, RT, in incongruent trials minus RT in congruent trials) is mostly interpreted to reflect the attentional focus on the response relevant information while ignoring response irrelevant information (see, e.g., C. W. Eriksen & St. James, 1986; White et al., 2011). This conclusion is based on the observation that the flanker congruency effect decreases with an increasing time between target and distractor onset (C. W. Eriksen & Schultz, 1979). When the distractors occurred at the same time as the target, the attentional focus was wide, leading to a larger congruency effect. On the other hand, in trials in which the distractors appeared after the target, the attentional focus was more narrow on the target, resulting in a smaller congruency effect (C. W. Eriksen & Schultz, 1979). Therefore, the flanker task seems suitable for testing the impact of hand-arm vibrations on the attentional focus. In addition, we will consider overall RTs and accuracy (independent of the flanker identity) as an indicator of general task performance.

## Possible impacts of hand-arm vibrations on the flanker effect

As knowledge on the impact of hand-arm vibrations on cognitive performance is limited, it is difficult to derive clear hypothesis on how hand-arm vibrations might impact on task performance in the flanker task. However, research studying other types of noise, as, e.g., visual or auditive noise, can provide useful analogies.

In the visual domain, Lavie and Tsal (1994) argue that perceptual load, the load of the perceptual system due to the processing of relevant information, is an important predictor of selective attention and the processing of irrelevant information. In her perceptual load theory, Lavie (1995) states that as long as perceptual capacity exists, attention is automatic and not subject to complete voluntary control. In cases with low perceptual load, irrelevant information can impact on the processing of relevant information. However, as soon as the perceptual capacity is exceeded (high load), attention is limited and less interference should be observed. In line with these predictions, she observed that irrelevant distractors interfere stronger in cases with low perceptual load, e.g., processing of only colors, than in cases with high perceptual load, e.g., processing of a conjunction of color and form, even when the stimulus screens are held completely constant across conditions (Lavie, 1995). However, cognitive load and response times are mostly correlated, such that higher cognitive load conditions often go along with longer response times. Thus, some argue that the difference in congruency effects are rather driven by a difference in response times than a difference in cognitive load (Miller, 1991). Most often, perceptual load is only defined as the load in visual perception. However, assuming a limited capacity of attention across all processes (Kahneman, 1973), the construct of load could also include other perceived sensory stimuli, such as vibration. Thus, if vibration adds perceptual load, we would expect response times to increase and the congruency effect to decrease in conditions with hand-arm vibrations compared to conditions without hand-arm vibrations.

In analogy to studies investigating the impact of noise, we would, however, expect a different pattern of results. Early studies on the impact of auditory noise revealed increased response time differences comparing incongruent to neutral stimuli in a Stroop task (Hartley and Adams, 1974, but see Smith and Broadbent, 1985, for no impact). These findings suggest increased interference and, thus, reduced selective attention under noise. Recently, a study using road traffic noise (Schlittmeier et al., 2015) replicated the finding of Hartley and Adams (1974) and observed increased Stroop congruency effects under noise compared to baseline conditions. A meta-analysis by Szalma and Hancock (2011) indicated that there was in general an effect of auditory noise on perceptual, cognitive, motor, and communication tasks, with speech noise inducing higher performance costs than non-speech noise. Furthermore, intermittent noise induced higher costs on performance than continuous noise. Thereby, the difference in intensity drives the effect and not intensity in itself.

Szalma and Hancock (2011) argue that the results of their meta-analysis are in line with the maximal adaptability theory from Hancock (1989). This theory states that individuals are fairly good at adjusting to stress, which can occur at the input level (e.g., due to noise), during the adaptive process, or at the output level. However, adaption fails at the extremes of underload and overload, which becomes evident by performance decrements. Thus, in analogy to the impact observed for auditory noise, one could expect that the experience of hand-arm vibrations might lead to increased congruency effects and decreased performance in general. In addition, one might expect a higher impact due to intermitted or changing vibration compared to constant vibration.

Based on these considerations, we investigated the impact of hand-arm vibrations on performance in a flanker task across three conditions: constant amplitude and frequency, randomly varied amplitude and frequency, and no vibration (control condition). If the transmitted hand-arm vibrations impact on attention in general and on the attentional focus specifically, we would expect to observe changes in mean RT as well as in the size of the flanker congruency effect. However, if hand-arm vibrations do not impact on attention or the attentional focus, we expect to observe neither changes in the mean RT nor changes in the flanker congruency effect.

## Experiment 1

### Methods

The pre-registration, all files to run the experiment, the participants’ data as well as the analysis scripts are available on the open science framework (OSF; https://osf.io/2f3ha/).

#### Participants

We collected data from 51 participants. Data of two participants had to be excluded due to technical problems and one participant aborted the experiment. None of the participants had to be excluded based on our other preregistered exclusion criteria, thus all participants answered at least 70% of the trials correctly, and none of the subjects’ arcsine transformed error rates or mean log transformed RTs deviated more than three SDs from the sample mean. This resulted in our preregistered sample size of 48 valid data sets. We chose 48 data sets as it was the minimum number that allowed the counterbalancing of all factors (order of conditions, response mapping). Collecting data of 48 participants allows us to detect medium sized effects of *f* = .24, with *α* = .05, 1–*β* = .95, in a measure repeated ANOVA with 1 group and 3 measurement time points, an estimated correlation between measures of .5, and a nonsphericity correction of *ϵ* = 1 according to G*Power (Faul et al., 2009). The included participants (9 male, 38 female, 1 diverse) were on average 20.65 years old (SD = 2.19) with a range of 18 to 32 years. We recruited participants via the online platform Sona-Systems University Freiburg. Participants had to be aged between 18 and 45 years, speak German well and needed normal or corrected-to-normal vision. All participants participated for partial course credit.

#### Material

**Figure 1:**
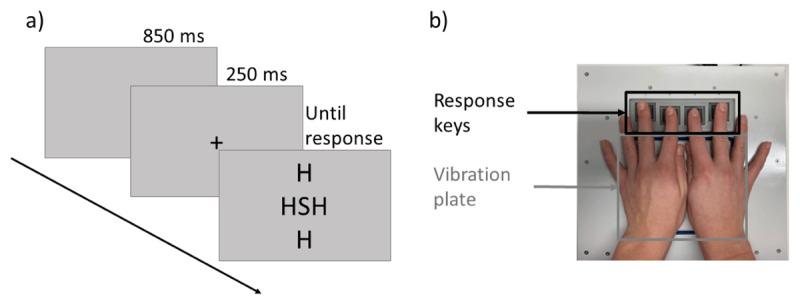
Trial sequence **(a)** and response keys and vibration platform **(b)** for Experiment 1.

We programmed the experiment using psychopy (Peirce et al., 2019). We used a vibration platform to stimulate both hands while the participants performed the flanker task (see Figure 1). The platform included a square plate with a side length of 13 cm that transmitted the vibration. Four response keys were positioned in front of the plate.

The stimuli consisted of the letters “H”, “S”, “F”, and “D”. All letters were presented in black on a gray screen at a viewing distance of 50–60 cm. The letter arrangement appeared on the screen with a size of 14 cm. In each trial, we presented arrangements of five letters at the center of the screen. The central letter of the arrangement served as the target letter, to which participants responded by pressing the corresponding key. Four identical distractor letters surrounded the target letter, which were located at the top and bottom as well as to the left and the right of the target.

#### Procedure

Before the actual experiment started, the participants signed an informed consent. Then, the instructions were presented: The participants were asked to place both hands in the instructed position on the vibrating plate, with their index and middle fingers on the corresponding response keys in front of the plate. Furthermore, participants were asked to avoid own body vibrations (e.g., bouncing legs).

The experiment was divided into four blocks. Each participant started with a practice block without vibration. The main experiment consisted of three different blocks: one without vibration, one with a constant frequency and amplitude of vibration, and one with a random frequency and amplitude of vibration. Block order was counterbalanced across participants. In the block with a constant frequency and amplitude of vibration, a frequency of 25 Hz and an amplitude of 8000 were administered.^1^ In the condition with random frequency and amplitude of vibration, the frequencies varied between 20, 25, 30, 35, and 50 Hz, and the amplitude varied between 6500, 7500, and 8000.

The practice block consisted of 34 trials. Each experimental block consisted of 200 trials with two additional warm-up trials at the beginning of each block. In both practice and experimental blocks, congruent and incongruent trials were equally frequent. Each trial comprised a target and four task-irrelevant distractors. The task-irrelevant distractors were either identical to the target letter (congruent stimuli) or different (incongruent stimuli). To avoid repetitions of stimuli and responses in consecutive trials, letters were paired such that the Letters “H” and “S” occurred as target and distractors in odd trial numbers whereas the letters “F” and “D” occurred in the remaining even trial numbers. The letters “H” and “S” and “F” and “D” were assigned either to the index fingers or the middle fingers of both hands. The letter to response key mapping was counterbalanced across participants and was presented during all trials at the bottom of the screen.

Each trial started with a black fixation cross presented for 250 ms in the center of the screen. This was followed by the target and the task-irrelevant distractors, which remained on screen until a response was recorded. A new trial started 850 ms after the response. In the practice block, additional error feedback was provided for 5 s, reminding participants of the letter assignment before the next trial started.

Participants were given time to study the mapping of letters to response keys before the practice block started. Participants were instructed to respond to each stimulus as fast and as accurately as possible. After each block, they received feedback about their average RT and the percentage of correct responses and were encouraged to respond faster in the next block without making more errors. Between two blocks, they had the possibility to take a short break.

After completing the three experimental blocks, participants took part in a short follow-up survey, which included two questions:

1. Did you use any strategies during the experiment? If so, which ones?
2. How would you asses the vibration and the difficulty of the experiment?

The duration of the experiment was approximately 30 min.

#### Analyses

We analyzed the data using R (R Core Team, 2016). To test our hypothesis, we conducted a 2 × 3 within-subject frequentistic ANOVA (afex package version 1.2-1) including the factors congruency (congruent, incongruent) and vibration condition (no vibration, constant amplitude and frequency of vibration, random amplitude and frequency of vibration). As dependent variable we considered RT and the arcsine transformed proportion of correct responses. As inference criteria we used *α* = .05.

In addition to the pre-registered analysis, we conducted multiple Bayesian ANOVAs using the package BayesFactor in R (version 0.9.12-4.6) with default priors. First, we used the same factors as in the frequentist version to compute how more likely the baseline model is compared to the models that included the effects we were interested in. Second, based on a reviewer suggestion, we computed a Bayesian ANOVA in which we additionally considered the factor congruency in trial *n*-1 to test for the impact of hand-arm vibrations on adjustment-related processing as for example indicated by the congruency sequence effect. And third, we used the same factors as in our first analysis but considered a dependent measure that integrates response times and accuracies. Therefore, we computed the balanced integration score, the difference between the *z*-standardized mean RT in correct responses and the *z*-standardized mean proportion of correct responses (for more information see Liesefeld & Janczyk, 2019; Liesefeld et al., 2015). For all Bayesian analyses we considered a model that included an intercept and the main effect of congruency as baseline model.

### Results

Prior to data analysis, we excluded trials from the practice block, the two warm-up trials at the beginning of each block, post-error trials (5.5% of total trials), and trials in which participants did not conduct the task properly, defined as extremely fast or slow responses (0.02% of total trials). Extremely slow responses were defined as responses slower than 3 s. To determine extremely fast responses, we computed the absolute and cumulative proportion of correct responses across participants in short spaced bins of 50 ms each. We used as the lower boundary the boundary of the first of two successive intervals that showed performance above chance level in both the absolute and cumulative accuracy. For the present data, this corresponded to a lower limit of 250 ms.

#### Analysis of response times

**Figure 2:**
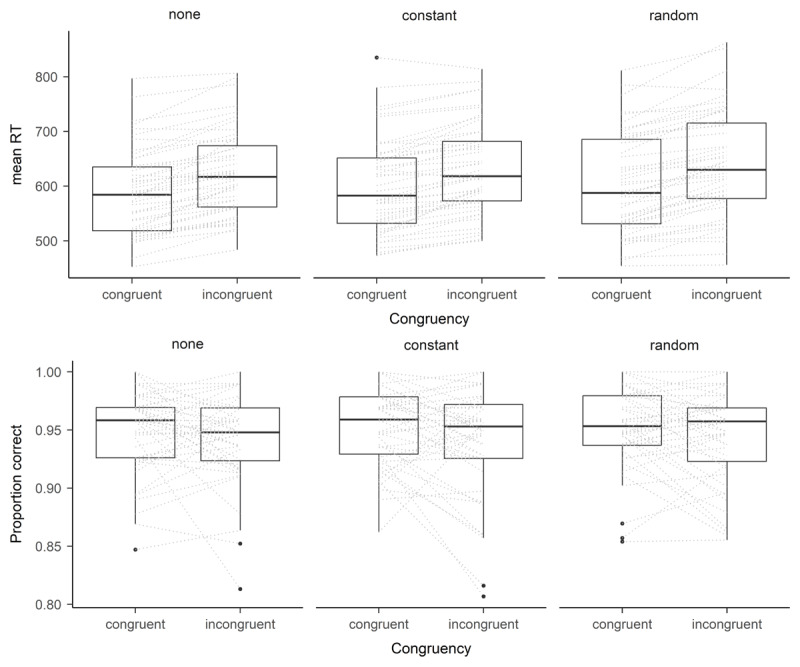
Experiment 1: Boxplot of mean response times (RT; upper panel) and proportion correct (lower panel) separately per vibration condition and congruency. *Note*. Dashed lines represent differences of individuals within one vibration condition.

For the analysis of mean RTs, we additionally excluded error trials. We conducted a 2 × 3 ANOVA with the within-subject factors congruency (congruent vs. incongruent) and vibration condition (constant vibration, random vibration, and no vibration). There was a significant main effect of congruency, *F*(1, 47) = 171.01, *p* < .001, \[
\eta _{p}^{2}\] = .784 (see Figure 2), indicating the flanker congruency effect with faster responses in congruent trials (*M* = 597 ms, *SE* = 79 ms) compared to incongruent trials (*M* = 632 ms, *SE* = 79 ms). Neither the main effect of vibration condition, *F*(2, 94) = 1.73, *p* = .184, \[
\eta _{p}^{2}\] = .035, nor the interaction between vibration condition and congruency, *F*(2, 94) = 1.33, *p* = .269, \[
\eta _{p}^{2}\] = .028, showed significant effects.

To investigate whether this absence of evidence is actually evidence of absence of an effect, namely that vibration condition does neither impact on mean RTs nor on the flanker congruency effect, we conducted an explorative Bayesian ANOVA using RT as dependent variable. We used a model with an intercept and the main effect of congruency as baseline model and tested whether there was evidence in favor of or against including a main effect of vibration condition or the interaction between vibration condition and congruency. Evidence was indecisive whether or not to include the effect of vibration condition, *BF*_1,0_ = 1.16, indicating that a model that included the main effect of vibration condition in addition to the effects of the baseline model was 1.16 times more likely than the baseline model. However, data provided evidence for excluding the interaction between congruency and vibration condition, *BF*_0,1_ = 12.53, indicating that the baseline model was 12.53 times more likely than a model that additionally included the interaction.

In addition, we exploratively examined the impact of vibration condition on adjustment-related processes such as congruency sequence effects (Gratton et al., 1992). Congruency sequence effects describe the observation that congruency effects in trial *n* are smaller after an incongruent stimulus in trial *n*-1 compared to a congruent stimulus in trial *n*-1. Therefore, we computed an additional Bayesian ANOVA using mean RT as dependent variable. Again, we used a model with an intercept and the main effect of congruency as baseline model and tested whether there was evidence in favor of or against including the interaction between congruency and *n*-1 congruency or including both the interaction between congruency and *n*-1 congruency and the three-way interaction between vibration condition, congruency and *n*-1 congruency. Data provided evidence for excluding the interaction between congruency and *n*-1 congruency, *BF*_0,1_ = 6.08. This indicates that the baseline model was 6.08 times more likely than a model that additionally included the interaction and, therefore, reveals the absence of a congruency sequence effect. Additionally, data provided strong evidence for the baseline model compared to a model that included both interactions, *BF*_0,1_ = 2.08 × 10^21^, indicating that the baseline model was 2.08 × 10^21^ times more likely than a model that additionally included both interactions. Therefore, the result reveals that there was no congruency sequence effect and no modulation of the congruency sequence effect by vibration condition.

#### Analysis of accuracies

As in the analyses for RTs, we conducted a 2 × 3 ANOVA with the within-subject factors congruency (congruent vs. incongruent) and vibration condition (constant vibration, random vibration, and no vibration). Again, there was a significant main effect of congruency, *F*(1, 47) = 7.17, *p* = .010, \[
\eta _{p}^{2}\] =.132 (see Figure 2) indicating the flanker congruency effect with more accurate responses in congruent trials (*M* = .952, *SD* = .030) compared to incongruent trials (*M* = .943, *SD* = .035). Neither the main effect of vibration condition, *F*(2, 94) = 0.30, *p* = .744, \[
\eta _{p}^{2}\] =.006, nor the interaction between vibration condition and congruency, *F*(2, 94) = 0.21, *p* = .812, \[
\eta _{p}^{2}\] =.004, showed significant effects.

Again, we conducted exploratively a Bayesian ANOVA using arcsine transformed performance correct as dependent variable to investigate whether there is no impact of vibration condition on accuracies or the flanker effect. In parallel with the analysis of RTs, we used a model with an intercept and the main effect of congruency as baseline model and tested whether there was evidence in favor of or against including a main effect of vibration condition or the interaction between vibration condition and congruency. Data provided evidence in favor of excluding the effect of vibration condition, *BF*_0,1_ = 21.22, indicating that the baseline model was 21.22 times more likely than a model that additionally included the main effect of vibration condition. Furthermore, data provided evidence for excluding the interaction between congruency and vibration condition, *BF*_0,1_ = 11.92, indicating that the baseline model was 11.92 times more likely than a model that additionally included the interaction.

We additionally computed exploratively a Bayesian ANOVA using arcsine transformed performance correct as dependent variable to investigate the impact of vibration condition on the congruency sequence effect in proportion of correct responses. Again, we used a model with an intercept and the main effect of congruency as baseline model and tested whether there was evidence in favor of or against including the interaction between congruency and *n*-1 congruency or including both the interaction between congruency and *n*-1 congruency and the three-way interaction between vibration condition, congruency and *n*-1 congruency. Data provided evidence for excluding the interaction between congruency and *n*-1 congruency, *BF*_0,1_ = 4.35. This indicates that the baseline model was 4.35 times more likely than a model that additionally included the interaction and, therefore, suggests the absence of congruency sequence effects. Additionally, data provided strong evidence for the baseline model compared to a model that included both interactions, *BF*_0,1_ = 148.5. This indicates that the baseline model was 148.5 times more likely than a model that additionally included both interactions and, therefore, reveals that the congruency sequence effect was not modulated by vibration condition.

#### Balanced integration score

To additionally check the robustness of the two separate analyses of RTs and accuracies and to correct for potential speed-accuracy tradeoffs, we computed an additional Bayesian ANOVA using the balanced integration score as dependent variable. The balanced integration score is the difference between the *z*-standardized RT of correct responses and the *z*-standardized proportion of correct responses (Liesefeld et al., 2015). Again we used a baseline model that included the intercept and the main effect of congruency and tested whether there was evidence in favor of or against a model including a main effect of vibration condition or the interaction between vibration condition and congruency. Data provided evidence in favor of excluding the effect of vibration condition, *BF*_0,1_ = 11.39, indicating that the baseline model was 11.39 times more likely than a model that included the main effect of vibration condition. Furthermore, data provided evidence for excluding the interaction between congruency and vibration condition, *BF*_0,1_ = 14.05, indicating that the baseline model was 14.05 times more likely than a model that additionally included the interaction.

### Discussion

In summary, we observed a flanker congruency effect in mean RTs and proportion of correct responses in all conditions. More importantly, neither the overall proportion of correct responses and the balanced integration score nor the flanker effect in RTs, accuracies, and the balanced integration score was modulated by the vibration condition. However, evidence was indecisive regarding the impact of hand-arm vibrations on mean RTs. In general, results of Experiment 1 indicate that hand-arm vibrations do not affect the attentional focus. However, the results on performance in the flanker task are quite surprising when considering the qualitative feedback given by participants. The majority of participants reported the vibration to be distracting in at least one of the conditions, while other participants reported it to be pleasant and even helpful. Therefore, in Experiment 2 we aimed to take a closer look at the discrepancy between the reported introspective experience and the objective performance in RTs and proportion of correct responses.

## Experiment 2

Thus, the aim of Experiment 2 was two-folded. First, we aimed to replicate the behavioral results from Experiment 1 using a different selective attention task. We decided to use a temporal version of the flanker task, because congruency effects in the temporal version of the flanker task tend to be larger than in the spatial version of the flanker task (e.g., C. W. Eriksen & Schultz, 1979; Hübner & Töbel, 2019; Wendt et al., 2014)^2^ In a temporal flanker task, a distractor precedes the target presentation. Therefore, it assesses the attention to a specific point in time rather than the width of the spatial attentional focus. In addition, we increased the number of trials per vibration condition from 200 to 300 trials and thus increased the duration of the exposure to each of the vibration conditions.

Second, we aimed to investigate whether the actual observed performance correlates with the subjective experience when performing the temporal flanker task. Regarding subjective experience, we investigated whether participants assess their own performance differently under vibration compared to no vibration (for examples on how introspective performance might differ to objective performance see, e.g., Bratzke & Bryce, 2022; Bryce & Bratzke, 2017). In addition, we explored whether the assessment of the vibration in terms of experienced vibration comfort and vibration discomfort correlates with the actual observed performance. Therefore, we included after blocks of 50 trials questions addressing the introspective concentration, introspective response speed, and introspective accuracy and in vibration blocks, additionally, experienced vibration comfort and discomfort.

Comfort and discomfort are two partially independent constructs (Zhang et al., 1996) that are often considered in research targeting ergonomics. While comfort is mostly associated with positive feelings, discomfort is associated with negative experiences (Bisht & Khan, 2013). As the affect signaling hypothesis (Dignath et al., 2020; Dreisbach & Fischer, 2015) assumes that affect is essential for conflict monitoring and adaptation, we thought it important to measure potential positive and negative affect induced by vibration and to test exploratively its impact on task performance (speed and accuracy).

### Methods

The general procedure as well as the vibration conditions were the same as in the previous experiment with the exception that we used a temporal flanker task, introduced introspective questions, and increased the number of trials slightly. Therefore, in the next sections we will only highlight these differences. The pre-registration, all files to run the experiment, the participants’ data as well as the analysis scripts are available on OSF (https://osf.io/2f3ha/).

#### Participants

We used a Bayesian sequential sampling procedure and started to collect a minimum of 30 valid data sets. Afterwards, we planned to continue sampling in case that evidence was still indecisive regarding whether to include i) the main effect of vibration condition in a baseline model that only included an intercept and the effect of congruency or ii) whether to include the interaction of vibration condition and congruency in the baseline model (for more details, see the analysis section). We planned to stop sampling as soon as the Bayes-Factor of both effects crossed the value of 5 or 1/5 for both arcsine transformed proportion of correct responses and RTs as dependent variables. This was already the case for 30 valid data sets. In total we collected data from 32 participants using the same inclusion criteria as in the previous study but additionally restricting the participant pool to participants who did not participate in Experiment 1. One participant had to be excluded due to having less than 70% correct responses. For one participant we had technical problems. This resulted in our final sample of 30 valid data sets (10 male, 20 female, 0 diverse). Participants were on average 23.13 years old (SD = 4.42) with a range of 19 to 37 years. Participants received either partial course credit or a monetary compensation.

#### Material

We used the same apparatus and letters as in the previous experiment. For the temporal flanker task, we presented always two letters consecutively at the screen center. The distractor letter appeared with a size of 2 cm on the screen. The target was smaller, with a size of 1.2 cm.

#### Procedure

Participants again worked through four blocks, one practice block and three experimental blocks. The practice block consisted of 42 trials. Each experimental block consisted of 300 trials. After each 50 trials, participants answered three to five introspective items. The first two trials at the beginning of each block, as well as those following each break for introspective questions, served as warm-up trials.

Each trial started with a black fixation cross presented for 250 ms in the center of the screen. This was followed by the task-irrelevant distractor for 139 ms. After a blank screen for 35 ms, the target appeared and remained in the center of the screen for 139 ms. A new trial began 750 ms after the participant’s response. In the practice block, additional error feedback was provided for 5 seconds, reminding participants of the letter assignment in case of an incorrect response before the next trial began. Distractor and target were presented one after the other in black on a gray screen at a viewing distance of 50–60 cm.

In the experimental blocks, after each 50 trials, participants answered three introspective items about their performance on a six-point scale via mouse click. The questions were:

- “How was your concentration in the last few trials?” (“very bad” to “very good”)
- “How would you rate your speed in pressing the response key in the last few trials?” (“very slow” to “very fast”)
- “How would you rate your accuracy in the last few trials?” (“very inaccurate” to “very accurate”)

In blocks with hand-arm vibrations, we further assessed whether vibration was experienced as unpleasant as well as pleasant. In line with the observation that vibration comfort and discomfort are partly independent constructs, we asked two questions:

- “How strong was the unpleasant feeling in your body caused by vibration?” (“not unpleasant” to “very unpleasant”)
- “How strong was the pleasant feeling in your body caused by vibration?” (“not pleasant” to “very pleasant”)

Apart from these changes, the procedure was the same as in Experiment 1. The duration of the whole experiment was approximately 50 min.

#### Analysis

To test our hypotheses, we conducted a Bayesian analysis of variance using the package BayesFactor in R (version 0.9.12-4.6) with default priors. As baseline model, we considered a model that included an intercept and the main effect of congruency (congruent, incongruent). We tested the baseline model against a model that included additionally the main effect of vibration condition (no vibration, constant amplitude and frequency of vibration, random amplitude and frequency of vibration). Furthermore, we tested the baseline model against a model that included additionally the interaction between congruency and vibration condition. As dependent variables, we considered both the mean RTs and the arcsine transformed accuracies.

In addition to the pre-registered analysis, we conducted based on a reviewer suggestion multiple Bayesian ANOVAs parallel to those in Experiment 1. We computed a Bayesian ANOVA in which we additionally considered the factor congruency in trial *n*-1 to test for the impact of hand-arm vibrations on adjustment-related processing indicated by the congruency sequence effect. In addition, we analyzed the balanced integration score, the difference between the *z*-standardized mean RT in correct responses and the *z*-standardized mean proportion of correct responses. For all Bayesian analyses, we considered a model that included an intercept and the main effect of congruency as baseline model.

### Results

Prior to data analysis, we excluded trials from the practice block, the two warm-up trials at the beginning of each block and after introspective questions, post-error trials (6.7% of total trials), and trials in which participants did not conduct the task properly (0.46% of total trials), defined as extremely fast or slow (>3 s) responses. We used the same procedure to determine the lower limit as in the previous experiment. For the present data this corresponded to a lower limit of 250 ms.

#### Analysis of response times

**Figure 3:**
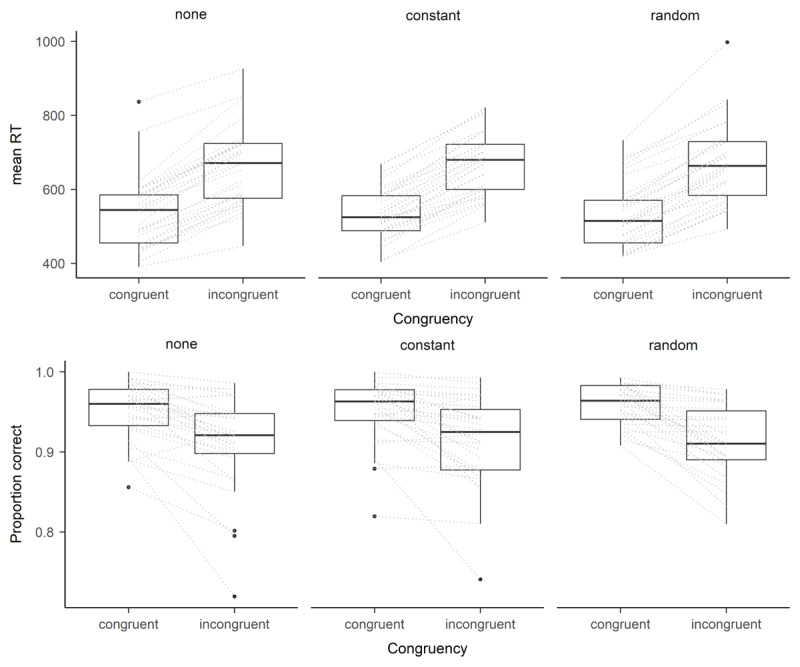
Experiment 2: Boxplot of mean response times (RT; upper panel) and proportion correct (lower panel) separately per vibration condition and congruency. *Note*. Dashed lines represent differences of individuals within one vibration condition.

For the analysis of RTs, we additionally excluded error trials. To investigate whether the experience of hand-arm vibrations interferes with RTs and the temporal flanker effect, we conducted a Bayesian ANOVA using RT as dependent variable. We used a model with an intercept and the main effect of congruency as baseline model and tested whether there was evidence in favor of or against including a main effect of vibration condition or the interaction between vibration condition and congruency. Our data showed descriptively the typical pattern of a temporal flanker effect with RTs being smaller in congruent trials (*M* = 535 ms, *SE* = 77 ms) than in incongruent trials (*M* = 669 ms, *SE* = 92 ms; see also Figure 3). Data provided evidence to exclude the effect of vibration condition, *BF*_0,1_ = 18.40, indicating that the baseline model was 18.40 times more likely than a model that included the main effect of vibration condition in addition to the effects of the baseline model. Thus, RTs did not differ between vibration conditions. Furthermore, data provided evidence for excluding the interaction between congruency and vibration condition, *BF*_0,1_ = 9.01, indicating that the baseline model was 9.01 times more likely than a model that additionally included the interaction. Therefore, vibration condition does not moderate the temporal flanker effect in RTs.

We additionally computed exploratively a Bayesian ANOVA using mean RT as dependent variable to investigate the impact of vibration condition on the congruency sequence effect in mean RTs. Again, we used a model with an intercept and the main effect of congruency as baseline model and tested whether there was evidence in favor of or against including the interaction between congruency and *n*-1 congruency or including both the interaction between congruency and *n*-1 congruency and the three-way interaction between vibration condition, congruency and *n*-1 congruency. Data provided evidence for including the interaction between congruency and *n*-1 congruency, *BF*_1,0_ = 6.41. This indicates that a model that additionally included the interaction was 6.41 times more likely than the baseline model, and, therefore, that a congruency sequence effect emerged in mean RTs in Experiment 2. Congruency effects were larger after a congruent (*M* = 147.8 ms) than after an incongruent (*M* = 118.6 ms) stimulus in trial n-1. However, data provided a strong evidence for the baseline model compared to a model that included both interactions, *BF*_0,1_ = 2.94 × 10^78^, indicating that the baseline model was 2.94 × 10^78^ times more likely than a model that additionally included both interactions. Thus, the congruency sequence effect seems not to be modulated by vibration condition.

#### Analysis of accuracies

To investigate whether the experience of hand-arm vibrations interferes with the proportion of correct responses and the temporal flanker effect, we conducted a Bayesian ANOVA using arcsine transformed proportion correct as dependent variable. Parallel to the analysis of RTs, we used a model with an intercept and the main effect of congruency as baseline model and tested whether there was evidence in favor of or against including a main effect of vibration condition or the interaction between vibration condition and congruency. Our data showed descriptively the typical pattern of a temporal flanker effect with a greater proportion of correct responses in congruent trials (*M* = .954, *SE* = .030) compared to incongruent trials (*M* = .914, *SE* = .045; see also Figure 3). The results were similar to Experiment 1. Data provided evidence to exclude the main effect of vibration condition, *BF*_0,1_ = 16.58, indicating that the baseline model was 16.58 times more likely than a model that included the main effect of vibration condition in addition to the effects of the baseline model. Thus, the proportion of correct responses did not differ between vibration conditions. Furthermore, data provided evidence for excluding the interaction between congruency and vibration condition, *BF*_0,1_ = 7.26, indicating that the baseline model was 7.26 times more likely than a model that additionally included the interaction. Therefore, vibration condition did not moderate the temporal flanker effect in accuracies.

We additionally computed exploratively a Bayesian ANOVA using arcsine transformed performance correct as dependent variable to investigate the impact of vibration condition on the congruency sequence effect in the proportion of correct responses. Again, we used a model with an intercept and the main effect of congruency as baseline model and tested whether there was evidence in favor of or against including the interaction between congruency and *n*-1 congruency or including both the interaction between congruency and *n*-1 congruency and the interaction between vibration condition, congruency and *n*-1 congruency. Data provided evidence for excluding the interaction between congruency and *n*-1 congruency, *BF*_0,1_ = 6.34. This indicates that the baseline model was 6.34 times more likely than a model that additionally included the interaction and, thus, that the congruency sequence effect did not emerge in the accuracy rates. Additionally, data provided strong evidence for the baseline model compared to a model that included both interactions, *BF*_0,1_ = 1.63 × 10^20^. This indicates that the baseline model was 1.63 × 10^20^ times more likely than a model that additionally included both interactions and, therefore, that the congruency sequence effect was not modulated by vibration condition.

#### Balanced integration score

Additionally, we computed a Bayesian ANOVA using the balanced integration score as dependent variable. Again we used a baseline model that included the intercept and the main effect of congruency and tested whether there was evidence in favor of or against including a main effect of vibration condition or the interaction between vibration condition and congruency. Data provided evidence in favor of excluding the effect of vibration condition, *BF*_0,1_ = 12.23, indicating that the baseline model was 12.23 times more likely than a model that included the main effect of vibration condition. Furthermore, data provided evidence for excluding the interaction between congruency and vibration condition, *BF*_0,1_ = 5.78, indicating that the baseline model was 5.78 times more likely than a model that additionally included the interaction.

#### Analyses of introspective items

To investigate whether there exists a discrepancy between the objectively observed behavior and the subjective perceived behavior, we conducted three separate analysis. First, we investigated whether the items measure different introspective constructs. Therefore, we computed the correlation between introspective concentration, introspective response speed, and introspective accuracy across all conditions and the correlations between introspective concentration, introspective response speed, introspective accuracy, experienced vibration comfort, and experienced vibration discomfort across conditions with constant and random vibration. Second, we inspected descriptively and using ANOVAs whether introspective concentration, introspective response speed, introspective accuracy, experienced vibration comfort, and experienced vibration discomfort difference between vibration conditions. And third, we tested whether vibration comfort and vibration discomfort are correlated with the actual observed performance. Therefore, we computed a linear mixed model using mean response time and mean error rate per sub-block as dependent variable.

##### Correlations between introspective items

**Figure 4:**
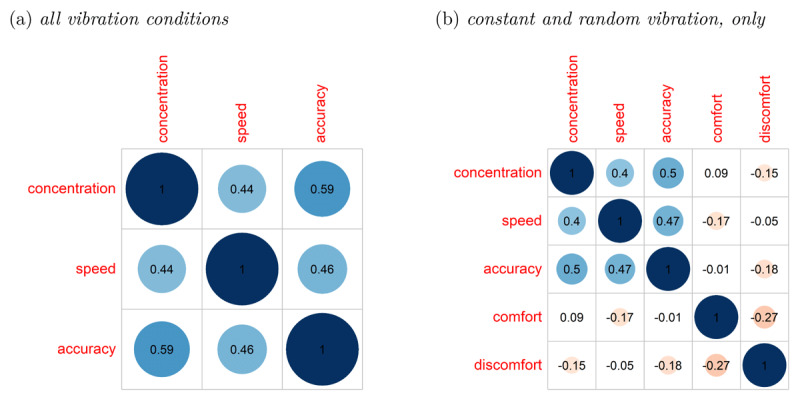
Correlation of introspective concentration, speed, accuracy, comfort, and discomfort. *Note*. Panel **(a)** depicts the correlation of introspective concentration, speed, and accuracy across all vibration conditions. Panel **(b)** considers the correlations only based on the data of the conditions with constant and random vibration. The numbers represent the correlation coefficients, blue indicate positive correlations, red negative correlations, and blank cells depict non-significant correlations.

As can be seen in Figure 4a, there were significant positive correlations between introspective concentration, speed, and accuracy across all vibration conditions (all *p*s < .001). Higher values in concentration went along with a higher perceived speed and a higher perceived accuracy. Furthermore, a higher perceived speed went along with a higher perceived accuracy. As the questions about vibration comfort and discomfort were only asked in the vibration condition with constant vibration and with random vibration, Figure 4b only includes the correlations of these two conditions. As can be seen, the correlation pattern between introspective concentration, speed, and accuracy is pretty similar to the correlation pattern across all vibration conditions. Additionally, vibration comfort is negatively correlated with vibration discomfort (*p* < .001). Please note that this correlation is only moderate, in line with the assumption that comfort and discomfort measure partially different aspects of vibration. Furthermore, introspective concentration, speed, and accuracy seem to be qualitatively different from comfort and discomfort as the correlations are rather low and most are not significant (although some significant correlations exist: concentration – discomfort, *p* = .004; comfort – speed, *p* = .002; accuracy – discomfort, *p* < .001).

##### Differences in introspective items between vibration conditions and sub-blocks

**Figure 5:**
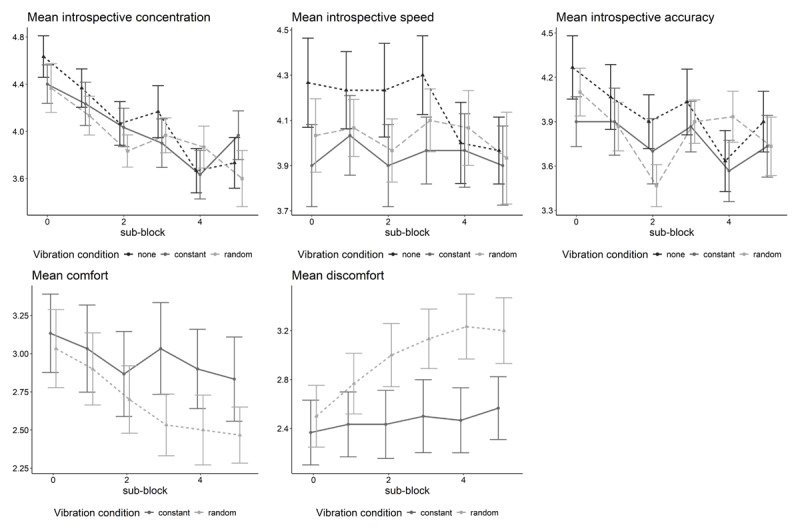
Mean introspective concentration, speed, accuracy, comfort, and discomfort separately for each block of 50 trials (sub-block) and vibration condition.

Figure 5 depicts the mean introspective concentration, speed, and accuracy as well as experienced vibration comfort and discomfort separately for each sub-block of 50 trials. As can be seen, introspective concentration, speed, and accuracy tend to decrease over sub-blocks, although this is only significant for introspective concentration and accuracy (see Table 1 for the test results of the ANOVA). Furthermore, there are descriptively only small and non-significant differences in introspective concentration, speed, and accuracy across vibration conditions. In contrast, descriptively there seems to be a difference in the perceived comfort and discomfort across vibration conditions which, however, is only significant for vibration discomfort. Participants perceived on average higher discomfort in the random vibration condition compared to the constant vibration condition. Additionally, there is a (significant) decrease of comfort and an increase of discomfort over time for both conditions.

**Table 1:** Test results from ANOVAs with the within-subject factors vibration condition and sub-block and introspective concentration, introspective speed, introspective accuracy, vibration comfort, and vibration discomfort as dependent variables.


	INTROSPECTIVE CONCENTRATION	

vibration condition	*F*(2, 57.7) = 0.43*p* = .654, \[ \eta _{p}^{2}\] = .014	

sub-block	***F*(2.3, 66.3) = 10.1*p* < .001, \[ \eta _{p}^{2}\] = .258**	

vibration condition × sub-block	*F*(6.1, 177.5) = 1.16*p* = .332, \[ \eta _{p}^{2}\] = .038	

	**INTROSPECTIVE SPEED**	**INTROSPECTIVE ACCURACY**

vibration condition	*F*(1.8, 51.3) = 2.17*p* = .130, \[ \eta _{p}^{2}\] = .070	*F*(1.9, 55.1) = 1.16*p* = .320, \[ \eta _{p}^{2}\] = .038

sub-block	*F*(3.3, 95.8) = 1.35*p* = .262, \[ \eta _{p}^{2}\] = .044	***F*(4.1, 120.2) = 3.55*p* = .008, \[ \eta _{p}^{2}\] = .109**

vibration condition × sub-block	*F*(6.3, 182.4) = 0.73*p* = .633, \[ \eta _{p}^{2}\] = .025	*F*(6.9, 200.7) = 0.80*p* = .584, \[ \eta _{p}^{2}\] = .027

	**VIBRATION COMFORT**	**VIBRATION DISCOMFORT**

vibration condition	*F*(1, 29) = 3.13*p* = .087, \[ \eta _{p}^{2}\] = .097	***F*(1, 29) = 6.99*p* = .013, \[ \eta _{p}^{2}\] = .194**

sub-block	***F*(3.1, 90.3) = 4.58*p* = .004, \[ \eta _{p}^{2}\] = .136**	***F*(3.3, 96.9) = 3.02*p* = .029, \[ \eta _{p}^{2}\] = .094**

vibration condition × sub-block	*F*(3.1, 88.7) = 0.98*p* = .405, \[ \eta _{p}^{2}\] = .033	*F*(3.9, 113.2) = 1.45*p* = .224, \[ \eta _{p}^{2}\] = .048


*Note*. The factor vibration condition includes no-vibration, constant vibration, and random vibration in the ANOVA for introspective concentration, speed, and accuracy. For vibration comfort and discomfort it includes only constant vibration and random vibration.

##### Relation between experienced vibration comfort and discomfort and actual task performance

To exploratively investigate the correlation between experienced vibration comfort and discomfort and mean RT and arcsine transformed accuracy, we conducted two separate linear mixed effect models in afex using REML and the Kenward-Roger approximation to derive *p*-values (as it is suggested in Luke, 2017). We considered comfort, discomfort, vibration condition (constant = 0, random = 1), the sub-block as well as the interaction between comfort and vibration condition and discomfort and vibration condition as predictors using participant ID as random intercept. We included sub-block as predictor as response times and error rates might change over time due to practice or fatigue.

**Figure 6:**
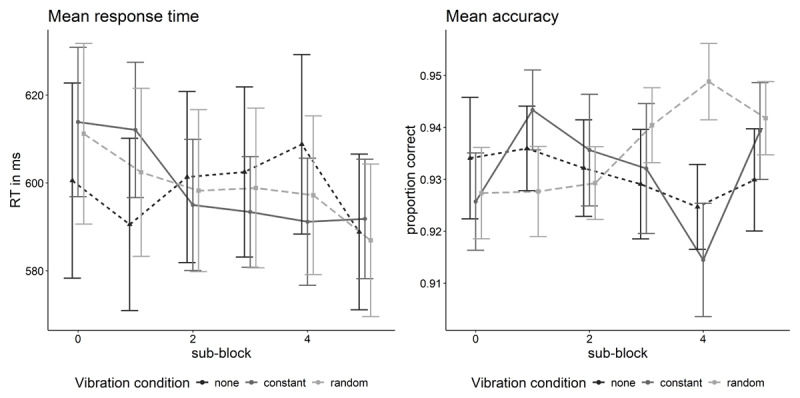
Mean speed and accuracy separately for each block of 50 trials (sub-block) and per vibration condition.

In line with the Bayesian ANOVA, there was no significant difference between vibration conditions in mean RT, *β* = –5.29, *t*(326) = –0.31, *p* = .759. But there was a significant effect of vibration comfort, *β* = 15.49, *t*(352) = 3.98, *p* < .001. As the constant vibration condition was coded with zero, this effect indicates that higher levels in comfort went along with higher mean RTs in the constant vibration condition. Additionally, the interaction between vibration comfort and vibration condition was also significant, *β* = –8.48, *t*(325) = 2.18, *p* = .030, indicating that within the random vibration condition, comfort had a less strong impact on mean RTs as within the condition with constant vibration. There was no significant effect of vibration discomfort (for the constant vibration condition), *β* = –5.48, *t*(346) = –1.56, *p* = .120. However, the interaction between vibration discomfort and vibration condition was significant, *β* = 11.70, *t*(326) = 3.16, *p* = .002, indicating that the vibration conditions differed significantly regarding the impact of experienced discomfort on mean RTs. Furthermore, there was a significant effect of sub-block *β* = –4.00, *t*(325) = 2.77, *p* = .006 indicating a speeding of responses in the later blocks (see Figure 6).

In contrast to the results of the Bayesian ANOVA, in the mixed linear model with arcsine proportion correct as criterion there was a significant effect of vibration condition, *β* = –0.10, *t*(330) = 3.19, *p* = .002, indicating that there were lower accuracies in the condition with random vibration compared to the condition with constant vibration. There was neither a main effect of vibration comfort, *β* = 0.01, *t*(291) = 1.45, *p* = .149, nor an interaction between vibration comfort and vibration condition, *β* = 0.01, *t*(330) = 1.50, *p* = .134. However, there was a significant effect of vibration discomfort, *β* = –0.02, *t*(338) = 3.32, *p* = .001, indicating that in the condition with constant vibration lower levels in discomfort went along with a higher accuracy. The interaction between vibration discomfort and vibration condition was also significant, *β* = 0.03, *t*(332) = 4.16, *p* < .001. This indicates that there was a significant difference in the relationship between discomfort on accuracies with the random vibration condition actually showing a positive relationship between discomfort and accuracy meaning that higher discomfort went along with higher accuracy. Furthermore, there was a significant effect of sub-block, *β* = 0.01, *t*(37) = 2.42, *p* = .016, indicating a higher accuracy in the later blocks (see Figure 6).

### Discussion

In Experiment 2, we were able to replicate the results from Experiment 1. The present data indicate evidence in favor of no effect of vibration on mean RTs, proportion of correct responses, and the balanced integration score and no effect on the temporal flanker effect in mean RTs, accuracies, and the balanced integration score, as well as no effect on the congruency sequence effect in mean RT or the proportion of correct responses.

Regarding introspective performance there were medium to high correlations between introspective concentration, speed, and accuracy. Whereas in line with the assumption that comfort and discomfort are partially independent constructs, vibration comfort and discomfort showed only small to medium correlations. Furthermore, neither introspective performance nor experienced vibration comfort differed across vibration conditions. However, vibration discomfort was rated higher in the condition with random vibration compared to the condition with constant vibration.

Further explorative analyses suggest that experienced vibration comfort correlates with mean RTs whereas discomfort correlates with accuracies in the temporal flanker task. For the condition with constant vibration, a higher experienced comfort went along with higher mean RTs, whereas a higher experienced discomfort went along with higher levels of accuracies. However, both of these effects were moderated by vibration condition. Interestingly, vibration comfort was also significantly correlated with introspective speed and vibration discomfort was significantly correlated with introspective accuracy. This might hint that participants have a quite good perception of their performance.

## General Discussion

To summarize, in the present study we were interested in whether the experience of hand-arm vibrations impacts on task performance and the attentional focus. In two experiments, we found neither an effect of random or constant hand-arm vibrations on the mean RT nor on proportion correct nor on an integrated measure of RT and proportion correct, the balanced integration score, in a flanker task and a temporal flanker task. Furthermore, there was no effect of hand-arm vibrations on the flanker effect and temporal flanker effect in mean RTs, accuracies, and the balanced integration score and no effect of hand-arm vibrations on the congruency sequence effect. In Experiment 2, Bayesian analyses even showed evidence in favor of no effect of hand-arm vibrations on mean RTs, accuracies the balanced integration score as well as the temporal flanker effect. These results indicate that the experience of hand-arm vibrations neither disrupts cognitive task performance in general nor the attentional focus or attention adjustment-related processes in specific.

The absence of an effect of hand-arm vibrations on the attentional focus is good news for the design of closely coupled human-machine interaction systems, given that in many tasks, in which hand-arm vibrations occur, they cannot be avoided. Nevertheless, these results are surprising with regard to the theories mentioned in the introduction. Based on both, perceptual load theory (Lavie, 1995) as well as previous studies on the impact of auditory noise (Schlittmeier et al., 2015; Szalma & Hancock, 2011), we would have at least expected to observe a difference in mean RT or accuracy. However, in both experiments we did not observe such an impact. Additionally, we did neither observe a better attentional focus as expected under the perceptual load theory (Lavie, 1995) nor a broadened attentional focus as expected based on studies about auditory noise (Hartley & Adams, 1974). Therefore, hand-arm vibrations seem to be qualitatively different to perceptual and auditory noise with regard to their impact on cognitive functions.

One reason why we have not observed any impact of hand-arm vibrations on the attentional focus might be due to the relatively short exposure times. For example, studies investigating the impact of auditory noise demonstrated that the duration of the noise can have an impact on the performance (Hartley & Adams, 1974; Smith & Broadbent, 1985). In our studies, participants were only exposed to hand-arm vibrations for approximately 10 min per vibration condition. One might argue that if the time of the vibration duration is an important predictor for cognitive performance, there should be a difference in performance across the sub-blocks and vibration condition. Neither of which we can observe in Figure 6. Rather performance seems to be quite constant across conditions throughout all time points. Yet, we cannot rule out that longer exposures to vibration might impact on cognitive processing. Please note, that the amount of vibration exposure that the participants received in the two blocks amounts to approximately 3.8 *m/s*^2^ and thus is rather close to the daily exposure dose of 5 *m/s*^2^ (according to Richtlinie 2002/44/EG des Europäischen Parlaments und des Rates vom 25. Juni 2002 über Mindestvorschriften zum Schutz von Sicherheit und Gesundheit der Arbeitnehmer vor der Gefährdung durch physikalische Einwirkungen (Vibrationen) (16. Einzelrichtlinie im Sinne des Artikels 16 Absatz 1 der Richtlinie 89/391/EWG) – Gemeinsame Erklärung des Europäischen Parlaments und des Rates, 2002). However, it resembles the vibration acceleration when working with, e.g., a cordless screwdriver or an angle grinder. Thus, we conjecture that the total amount of vibration exposure is sufficient to mimic actual vibration conditions in working environments.

Following the dynamic model of stress and sustained attention (Hancock, 1989), stress only impacts performance on high underload or overload. Therefore, in light of this model it doesn’t mean that participants do not show any performance decrease in general when experiencing hand-arm vibration. It just seemed that they were very good in adapting to the stress induced by the vibration we induced in the present study. Therefore, future research should investigate whether participants are still well able to adjust to stronger vibrations or longer vibrations or whether in such situations they cross the zone of maximal adaptability, postulated by Hancock (1989), which would lead to performance decreases. Alternatively, future studies might consider the impact of vibration on task performance in tasks that are more attention demanding. As can be seen in Figures 2 and 3 accuracy rates are quite high (close to ceiling effects) in both experiments. Thus, considering tasks that are more attention demanding might harm participants’ ability to adapt to the additional stress induced by vibration and thus might lead to a decreased performance.

Although we did not observe an impact of hand-arm vibrations on cognitive task performance, we were able to see first indicators of a relationship between vibration comfort and discomfort and performance. Based on the results of the explorative mixed linear model, vibration comfort seems to be a predictor for response speed whereas vibration discomfort seems to be a predictor for accuracy. This is interesting with regard to the design of, e.g., power tools, as vibration comfort and discomfort do not only change over time but also depend on vibration frequency (Fotler et al., 2024). Thus, filters can be construed that adapt vibration frequencies in such a way that performance is optimally supported which is especially interesting in the light of achieving human-machine symbiosis for dynamically changing systems (Inga et al., 2023). Therefore, it would be interesting to see whether this relationship replicates in future studies such that it could be considered as a design feature in future tools.

## Public significance

Hand-arm vibrations arise in a number of tasks which include power tools use or holding a workpiece that is currently under construction. Most of these tasks can neither be automated nor can the hand-arm vibrations be avoided. To achieve best work-performance and avoid accidents it is, therefore, important to know whether and under which circumstances they impact on selective attention to develop possible necessary corrective actions.

## Data Accessibility Statement

We preregistered the methods and analysis of the study on OSF. All materials to run the experiments and the data can be retrieved from OSF (https://osf.io/2f3ha/).

## Footnotes

Please note that due to the construction of the vibration platform the amplitude does not directly convert onto the metric scale. However, having an amplitude of 8000 on the vibration platform with no weight on it is equivalent to an amplitude of 1 mm. We used these numbers to compute the vibration exposure mentioned in the Discussion.

Please note that this task setting is also framed as prime-target task in many studies. In this case, distractor stimuli are termed prime stimuli (that can be congruent or incongruent to the target stimulus). Here, we decided for the wording “temporal flanker task” for reasons of consistency with Experiment 1.
